# Regulation of Mat Responses by a Differentiation MAPK Pathway in *Saccharomyces cerevisiae*

**DOI:** 10.1371/journal.pone.0032294

**Published:** 2012-04-04

**Authors:** Sheelarani Karunanithi, Jyoti Joshi, Colin Chavel, Barbara Birkaya, Laura Grell, Paul J. Cullen

**Affiliations:** Department of Biological Sciences, State University of New York-Buffalo, Buffalo, New York, United States of America; University of Minnesota, United States of America

## Abstract

Fungal species exhibit diverse behaviors when presented with extracellular challenges. Pathogenic fungi can undergo cell differentiation and biofilm formation in response to fluctuating nutrient levels, and these responses are required for virulence. In the model fungal eukaryote *Saccharomyces cerevisiae*, nutrient limitation induces filamentous growth and biofilm/mat formation. Both responses require the same signal transduction (MAPK) pathway and the same cell adhesion molecule (Flo11) but have been studied under different conditions. We found that filamentous growth and mat formation are aspects of a related response that is regulated by the MAPK pathway. Cells in yeast-form mats differentiated into pseudohyphae in response to nutrient limitation. The MAPK pathway regulated mat expansion (in the plane of the XY-axis) and substrate invasion (downward in the plane of the Z-axis), which optimized the mat's response to extracellular nutrient levels. The MAPK pathway also regulated an upward growth pattern (in the plane of the Z-axis) in response to nutrient limitation and changes in surface rigidity. Upward growth allowed for another level of mat responsiveness and resembled a type of colonial chemorepulsion. Together our results show that signaling pathways play critical roles in regulating social behaviors in which fungal cells participate. Signaling pathways may regulate similar processes in pathogens, whose highly nuanced responses are required for virulence.

## Introduction

Microbial species are single-celled organisms that typically grow and divide independently of other cells. In many species, congregations of microbial cells, called mats or biofilms, can adhere to surfaces and each other in specialized structures. A biofilm can be defined as the growth of a mat of microbial cells whose expansion or architecture is regulated by cellular decision-making pathways and cell adhesion molecules [1]. Biofilms can produce an extracellular matrix that contains polysaccharides, proteins and DNA [2]. Cells in a biofilm can undergo cell differentiation [1], communicate with other cells (quorum sensing, [3]), and regulate the growth of the mat [1]. These complex properties resemble in some ways the behaviors of multi-cellular organisms, and such “community living” is thought to provide a selective advantage to its members. For example, cells in a biofilm can be protected from extracellular stresses such as anti-microbial agents. Indeed, a new and complex picture of social evolution is emerging in microbial species [4], which is revolutionizing the otherwise simplistic view of a microbial “colony”.

Biofilms formed by pathogens are an important part of the threat posed by nosocomial infections. The major human fungal pathogen *Candida albicans* can grow in biofilms, which facilitates adherence to medical devices [5]. Candidal biofilms are complex spatiotemporally regulated structures [6] that consist of different cell types, including yeast, hyphal, and pseudohyphal forms [7], [8]. The cellular differentiation to filamentous growth in *C. albicans* and other pathogens occurs in response to nutrient limitation and other cues [9], [10], [11], [12], [13] and is itself required for virulence [14]. Hyphae contribute to the formation of biofilms in *C. albicans* [15], [16] and *Candida parapilosis* [17]. In *C. albicans*, The Cek1 mitogen activated protein kinase (MAPK) pathway is one of the several pathways that controls hyphal development [18] although its role in biofilm formation has not been as clearly established [15]. Likewise, in *C. parapilosis*, the cell-wall integrity MAPK Mkc1 is required for hyphal development and plays a role in biofim formation [19]. Among the changes regulated by signaling pathways is transcriptional regulation of genes that encode cell adhesion molecules [13], [20] like Als3 [21], which promote the adherence of cells to other cells and to surfaces.

The budding yeast *Saccharomyces cerevisiae* provides an attractive system to understand the genetic basis of fungal foraging behaviors. In response to nutrient limitation, *S. cerevisiae* can undergo filamentous growth, in which cells form pseudohyphae that invade into substrates [22]. *S. cerevisiae* can also form highly structured mats that adhere to agar and plastic surfaces [23]. Several findings suggest that filamentous growth and mat formation might be related in *S. cerevisiae*. Both responses share some of the same regulatory proteins, including Rim101 [24], [25], Opi1 [26], Ras2 [27], Yak1 [28], and Flo8 [29]. Both responses occur in response to nutrient limitation, including the dimorphic switch to pseudohyphae and the expansion and architecture of mats, characterized by a central hub and radiating spokes [22], [30]. Both responses are also thought to require the same signal transduction pathway, a MAPK pathway commonly referred to as the filamentous growth pathway [31], [32], [33], [34]. The filamentous growth pathway is controlled by the signaling mucin Msb2 [35], the cell-surface tetraspan protein Sho1 [35], [36], the Rho-GTPase Cdc42 and p21 activated kinase (PAK) Ste20 [37], [38], and a MAPK cascade that controls the activity of two transcription factors (Ste12 and Tec1, [39], [40], [41]). The filamentous growth pathway regulates the expression of many genes, including the major yeast cell adhesion molecule, Flo11, which is required for both filamentous growth [42], [43], [44] and mat expansion [23].

Under what conditions filamentous growth and mat formation may occur together in *S.cerevisiae*, and how they might be co-regulated, is not clear. One reason for this gap in understanding is that filamentous growth and mat formation have been studied under different conditions [7], [8]. Mat formation is studied on low-agar medium (YEPD+0.3% agar, [23]), in which mats expand on the surface in the plane of the XY-axis. Filamentous growth, on the other hand, is typically studied on standard agar medium (YEPD+2% agar, [32]) to assess the cells invading downward in the plane of the Z-axis. In the few cases that have been examined, cells in mats are mainly in the yeast-form cell type [23]. Some genes like vacuolar protein sorting genes [25] and *HSP70* [45] are specifically required for mat formation but not filamentous growth, which also suggests that the two responses have distinct genetic requirements.

In this study we address the relationship between filamentous growth and mat formation in *S. cerevisiae*. We show that filamentous growth and mat formation are part of an integrated behavior that is regulated by the MAPK pathway. Specifically, “filamentous mats” form under nutrient-limiting conditions, which exhibit characteristic mat properties (XY-axis expansion on semi-solid media and adherence to plastic) and filamentation responses (cell elongation, unipolar budding and invasive growth). Yeast mats also undergo an upward growth pattern that is regulated by the MAPK pathway. This growth pattern allows mats to establish boundaries with neighboring mats in a MAPK-dependent manner. Our study fills an important gap in understanding the regulation of nutritional foraging responses in *S. cerevisiae* and may generally extend to mat regulation in other fungal species including pathogens.

## Results

### S. cerevisiae Forms Filamentous Mats

**Figure 1:**
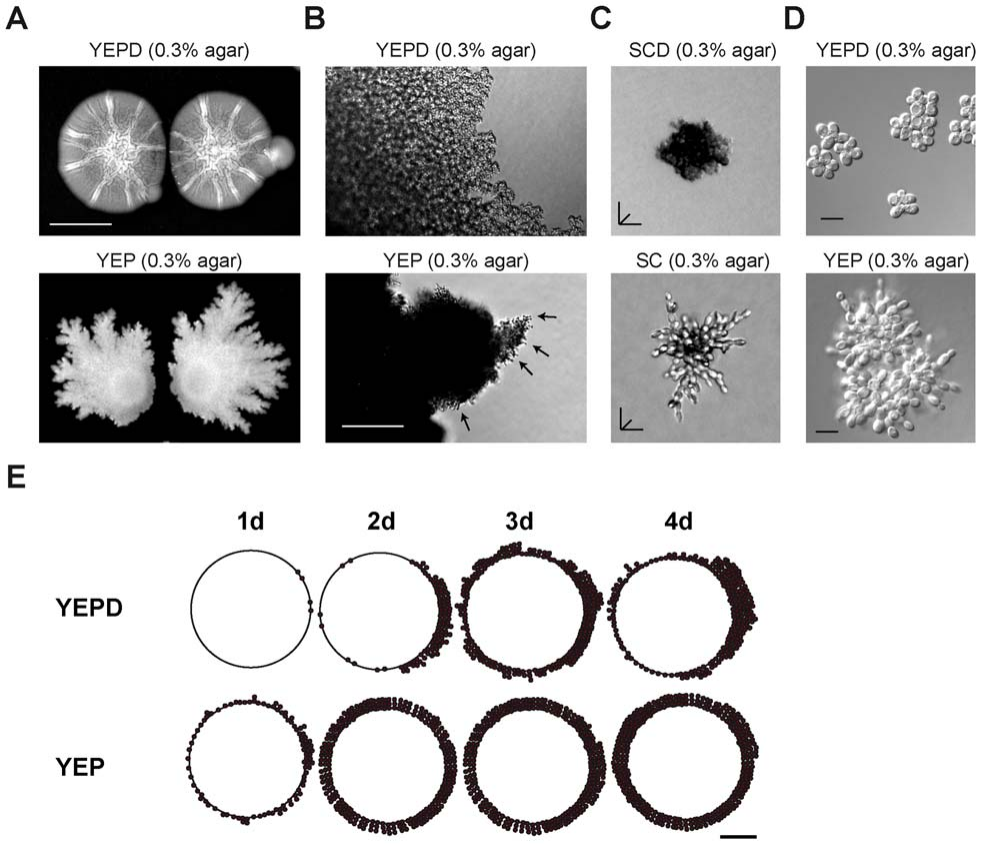
*S. cerevisiae* forms filamentous mats. **A**) Wild-type cells (PC313) were spotted 2 cm apart onto 0.3% agar media that contained (YEPD; top panel) or lacked (YEP; bottom panel) glucose. The YEPD plate was incubated for 4 days and photographed; the YEP plate for 15 days. Bar = 1 cm. **B**) Microscopic examination of perimeters of mats in 1A. Bar = 100 microns. **C**) The origin of filamentous mats. Wild type (PC538) cells were examined on synthetic medium either containing 2% glucose (SCD) or lacking glucose (SC) in 0.3% agar medium for 24 h at 30°C. A compiled Z-stack rendering of typical microcolonies are shown. Bar = 20 microns. **D**) Same strains in 1C were examined on rich medium either containing 2% glucose (YEPD) or lacking glucose (YEP) in 0.3% agar. A representative microscopic image is shown. Bar = 10 microns. E) Vegetative mats mature into filamentous mats over time as nutrients become limiting. Two mats of wild type (PC313) strain were spotted bilaterally (1.5 cm apart) on YEPD and YEP media (+0.2% galactose) containing 0.3% agar media. The number of filaments occurring along the circumference of mats was scored on a scale of 1, 2, or 3 dots at 20× magnification corresponding to 3, 6, or 9 filaments or greater, respectively. Dots were plotted on a circle representing the outline of one of the mats with right hemispheres corresponding to the side of the mat facing a second mat. Asymmetric filamentation observed in the right hemisphere of 2d, Glu can possibly result from nutritional stress compounded by nutrient depletion from adjacent mats. Filamentation was monitored and plotted after growth for 1, 2, 3, and 4 days. Quantitation of pseudohyphae was complicated at longer time points when biofilms began to variegate [60]. Bar = 1 cm.

To investigate the potential relationship between filamentous growth and mat formation, conditions were examined in which the two responses might coexist. Nutrient limitation induces filamentous growth [22], [30], and therefore mat formation was examined under nutrient-limiting conditions. Mats that formed in glucose-limiting conditions had a strikingly different appearance than mats that formed in glucose-rich conditions (Fig. 1A). Specifically, mats grown on glucose-limited media had a highly patterned periphery, with ruffled projections occurring at mat boundaries [Fig. 1A, YEP (0.3% agar)]. Microscopic examination showed the presence of filamentous cells along the periphery of mats grown in glucose-limited media [Fig. 1B, YEP (0.3% agar), arrows]. In contrast, mats grown in high-glucose media showed smooth perimeters [Fig. 1A, YEPD (0.3% agar)], which were composed of yeast-form cells [Fig. 1B, YEPD (0.3% agar)], as previously reported [23]. The single-cell invasive growth assay [30] showed that mats originating on high-glucose media were composed of yeast-form cells (Fig. 1, C and D, for both synthetic SCD media and YEPD, respectively), whereas mats that formed on glucose-limiting media were composed mostly of filamentous cells (Fig. 1, C and D, SC and YEP, respectively). Therefore, *S. cerevisiae* can form mats that contain different cell types.

**Figure 2:**
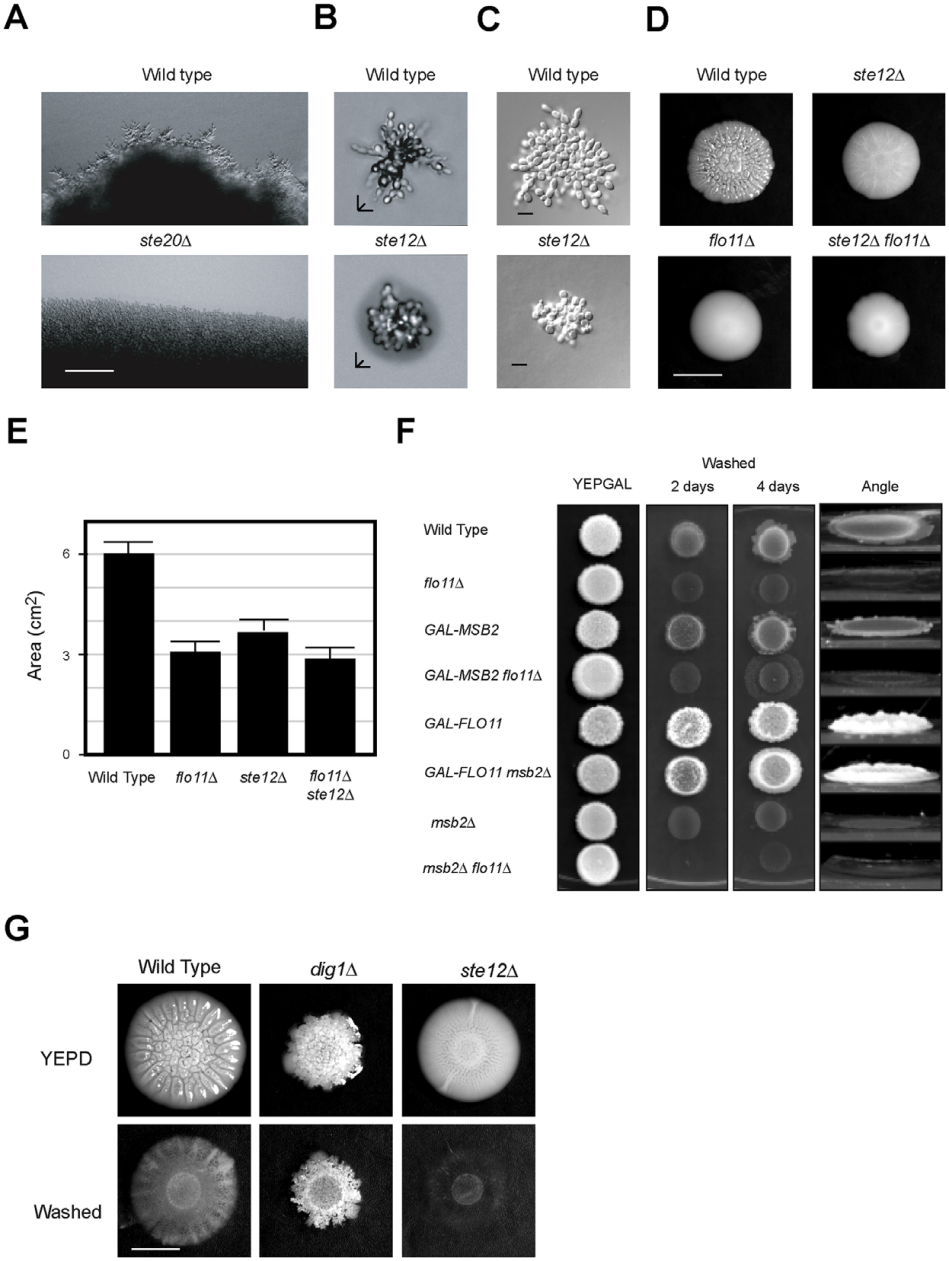
The filamentous growth pathway regulates foraging responses. **A**) Microscopic examination of mat perimeters grown on YEP 0.3% agar medium; top panel, wild-type cells (PC313), bottom panel *ste20*Δ mutant (PC549). Bar = 50 µM. **B**) The formation of pseudohyphae in filamentous mats is dependent on the MAPK pathway. Wild type (PC538) and the *ste12*Δ (PC539) mutant were spotted onto synthetic media (lacking glucose) in 0.3% agar medium for 24 h at 30°C. A compiled Z-stack rendering of typical microcolonies are shown. Bar = 10 microns. **C**) Same strains in 2B were examined on rich medium lacking glucose (YEP) in 0.3% agar. A representative microscopic image is shown. Bar = 10 microns. **D**) Genetic analysis of the roles of the MAPK pathway and Flo11 in mat formation. Cells of the indicated genetic backgrounds were spotted onto YEPD media (0.3% agar) for 4 days and a representative mat was photographed. Bar = 1 cm. **E**) Quantitation of the surface area of the mats in D. Mats were spotted in duplicate; error bars represent the average of two separate trials. **F**) Plate-washing assay of cells overexpressing *MSB2* and *FLO11* in various genetic backgrounds. Cells were spotted onto YEPGAL and incubated for 2d and 4d. The plate was photographed, washed, and photographed again. At right, a photograph of the plate taken at a 45° angle. **G**) Role of hyperactive MAPK pathway in regulating filamentous mats. Wild-type (PC538), *ste12*Δ (PC2382), *dig1*Δ (PC3039), were spotted onto YEPD media (0.3% agar) and grown at 30°C. Plates were photographed after 4d, cooled to 4°C for 30 min, washed and photographed again to reveal increase in invasion inhibits mat expansion. Bar = 1 cm.

In mats, yeast-form cells might differentiate into filamentous-form cells as nutrients become limiting. To test this possibility, mat perimeters were examined over time. In nutrient-rich conditions at early time points, mat perimeters were composed of yeast-form cells [(Fig. 1E, YEPD, 1d few/no filamentous cells (dots) on the mat surface (circle)]. Over time, mat perimeters became populated with filamentous cells (Fig. 1E, YEPD, 2d, 3d, and 4d, more dots). In contrast, mats that formed under nutrient-limiting conditions were composed of filamentous cells at early time points (Fig. 1E, YEP, 1d). Under both conditions, the filamentous cells along mat perimeters increased over time (Fig. 1E), presumably as nutrients became depleted. The data indicate that yeast-form mats mature into filamentous mats as nutrient levels decline. Filamentous growth allows the invasion into substrates [32]. A practical limitation to studying invasive growth of mats is that cells on low-agar media cannot be washed off of plates without disrupting the agar matrix. We performed the plate-washing assay on chilled (4°C for 30 min) 0.3% agar plates to maintain the integrity of the agar matrix, which showed that mats grown on low-agar media exhibit invasive growth (Fig. S1; and Fig. 2G, below). The invasive scar left by mats had characteristics of mat surface growth, including the central hub and radial spoke pattern. Microscopic examination showed that the invaded cells were a mixture of yeast and filamentous-form cells (data not shown). Therefore, mat formation and filamentous growth are aspects of an integrated nutrient-dependent foraging response.

### The Filamentous Growth Pathway Regulates Filamentous Mat Expansion

The filamentous growth pathway might regulate the formation of filamentous cells in mats. The filamentous growth pathway was required for filament formation at the perimeter of mats grown on glucose-limited (YEP) medium (Fig. 2A, *ste20*Δ). The filamentous growth pathway was also required for the formation of pseudohyphae in mat microcolonies (Fig. 2, B and C, *ste12*Δ). The *ste20*Δ, *ste12*Δ, and other MAPK mutants showed similar phenotypes. Accordingly, the filamentous growth pathway (*ste12*Δ) and its target Flo11 [29], [43], [46] were required for the invasive growth of mats (Fig. S1A).

The filamentous growth pathway is also thought to regulate mat formation, although its role in this process has not been fully investigated. We confirmed that the filamentous growth pathway is required for mat expansion (*ste12*Δ; Fig. 2D). Quantitation of mat area showed that the mat expansion defect of a *flo11*Δ mutant was similar to the *ste12*Δ single mutant and *flo11*Δ *ste12*Δ double mutant (Fig. 2E). Similarly, MAPK-dependent invasive growth required Flo11 (Fig. 2F). Specifically, overexpression of *MSB2*, which activates the filamentous growth pathway [35], stimulated agar invasion (Fig. 2F; *GAL-MSB2*) primarily through Flo11 (Fig. 2F; *GAL-MSB2 flo11*Δ). Some Flo11-independent agar invasion was observed (compare *msb2*Δ and *flo11*Δ single mutants to the *msb2*Δ *flo11*Δ double mutant, and the *GAL-MSB2* mutant to the *GAL-MSB2 flo11*Δ mutant) which might result from Flo11-independent aspects of invasive growth [such as unipolar budding and cell elongation [47]]. Overexpression of *FLO11* induced hyperinvasive growth (Fig. 2F; *GAL-FLO11*) that was independent of the MAPK pathway (Fig. 2F; *GAL-FLO11 msb2*Δ), in line with the idea that *FLO11* is a downstream target and a major regulator of the response [47].

We previously showed that modulating Flo11 adherence functions can optimize invasive growth and mat expansion, rather than maximize either response [48]. For example, increasing the ratio of cell-associated to shed Flo11 caused hyperinvasive growth while restricting mat expansion [48]. Given that the MAPK pathway regulates *FLO11* expression [29], [43], [46], we hypothesized that the MAPK pathway might similarly regulate mat expansion and invasive growth to optimize nutritional foraging. Consistent with this possibility, hyper-activation of the filamentous growth pathway, for example in cells lacking the transcriptional repressor Dig1 [49], [50], induced hyper-invasive growth (Washed *dig1*Δ, Fig. 2G) while inhibiting mat expansion (YEPD *dig1*Δ, Fig. 2G). Other mutants that hyper-activated the pathway showed a similar pattern (data not shown). Therefore, the filamentous growth pathway, along with other pathways, regulates *FLO11* expression to fine-tune nutritional foraging. We previously showed that the filamentous growth pathway exhibits a multimodal response, where the induction of *FLO11* expression occurs prior to dimorphism as nutrient levels decline (Pitoniak et al 2009). Thus in mats, the MAPK pathway may promote Flo11-dependent surface expansion (in the plane of the XY-axis), followed by differentiation-dependent invasive growth (downward in the plane of the Z-axis) in step with declining nutrient levels to optimize nutritional foraging.

Coordinating invasive growth and mat expansion might provide an advantage to cells engaged in this behavior. Indeed, surface expansion necessarily opens up new territories for invasive growth. Likewise, as shown below, pseudohyphae also contribute to mat architecture. We also tested whether filamentous projections serve as “sensors” to direct mat expansion along nutritional gradients, but found no evidence to support this possibility (data not shown). Therefore, the MAPK pathway, along with other signaling pathways that regulate *FLO11* expression [51], coordinate filamentous growth and mat expansion into an integrated response, which may function to optimize nutritional foraging.

### The Filamentous Growth Pathway Regulates the Upward Growth of Mats

**Figure 3:**
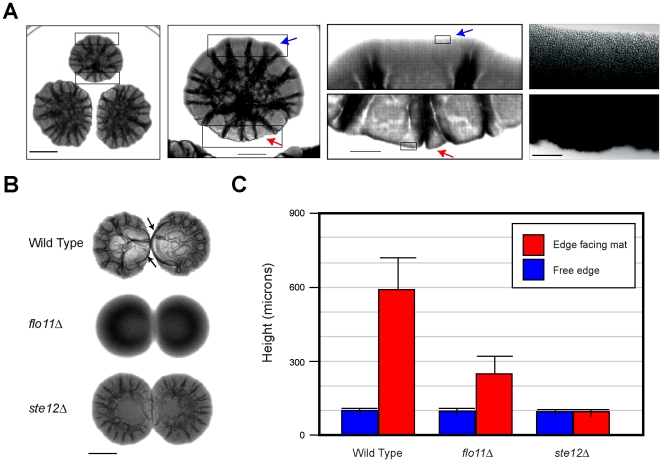
MAPK- and Flo11-dependent colony avoidance response. **A**) Wild-type cells were spotted in three spots and examined daily. The photograph showing the embossed appearance of colonies was taken at day 3. Left three panels, Bar = 1 cm. Far right panel, micrograph of cells at the perimeter of an asymmetrically forming biofilm. Bar, 200 microns. Mat borders facing (red arrows) or not facing (blue arrows) another mat are indicated. **B**) Wild type (PC538), *flo*11Δ (PC1029), and *ste*12Δ (PC2382) cells were grown on YEPD media for 18 h. Equal concentrations of cells were spotted, 1 cm apart, on to YEPD media containing 0.3% agar. Plates were incubated for 48 h at 30°C and photographed using transmitted light. Bar = 1 cm. **C**) Bar graph of height measurements (in mm) of the mat borders facing/not facing the adjacent mats on the right in B. Contour maps in the Z-axis of mats was generated. Seven readings after the first peak in the Z-axis were averaged to plot the graph. Standard deviation between measurements were used to generate the error bars.

In nutrient-limiting conditions, adjacent mats formed asymmetric patterns, which might be suggestive of a chemotropic behavior (Fig. 1A, -GLU). We found that mats encountering a neighboring mat grew upward in the plane of the Z-axis to form an elevated structure along the periphery (Fig. 3A). Microscopic examination showed that that the structures were compact elevated towers of cells (Fig. 3A, far right panels). Similarly, wild-type mats spotted bilaterally formed a rim-like structure (Fig. 3B, arrows) that was >5-fold taller when facing another mat (Fig. 3C, from ∼100 microns to >600 microns). This response was dependent on the MAPK pathway and Flo11. Specifically, the *ste12*Δ and *flo11*Δ mats expanded into each other (Fig. 3B) and did not show a dramatic height difference in response to the approach of an adjacent colony (Fig. 3C). Asymmetry likely results from nutrient depletion at one face of the mat relative to the other, based on the following reasons: it was not due to growth arrest on the side of the mat facing the adjacent colony, because microscopic examination showed that cells continued to form buds, and it was not the result of secreted molecules from adjacent colonies [52], because isolated mats that were exposed to nutrient gradients also formed asymmetric patterns (data not shown). The response might represent a type of chemorepulsion that would allow mats to establish boundaries from other mats.

**Figure 4:**
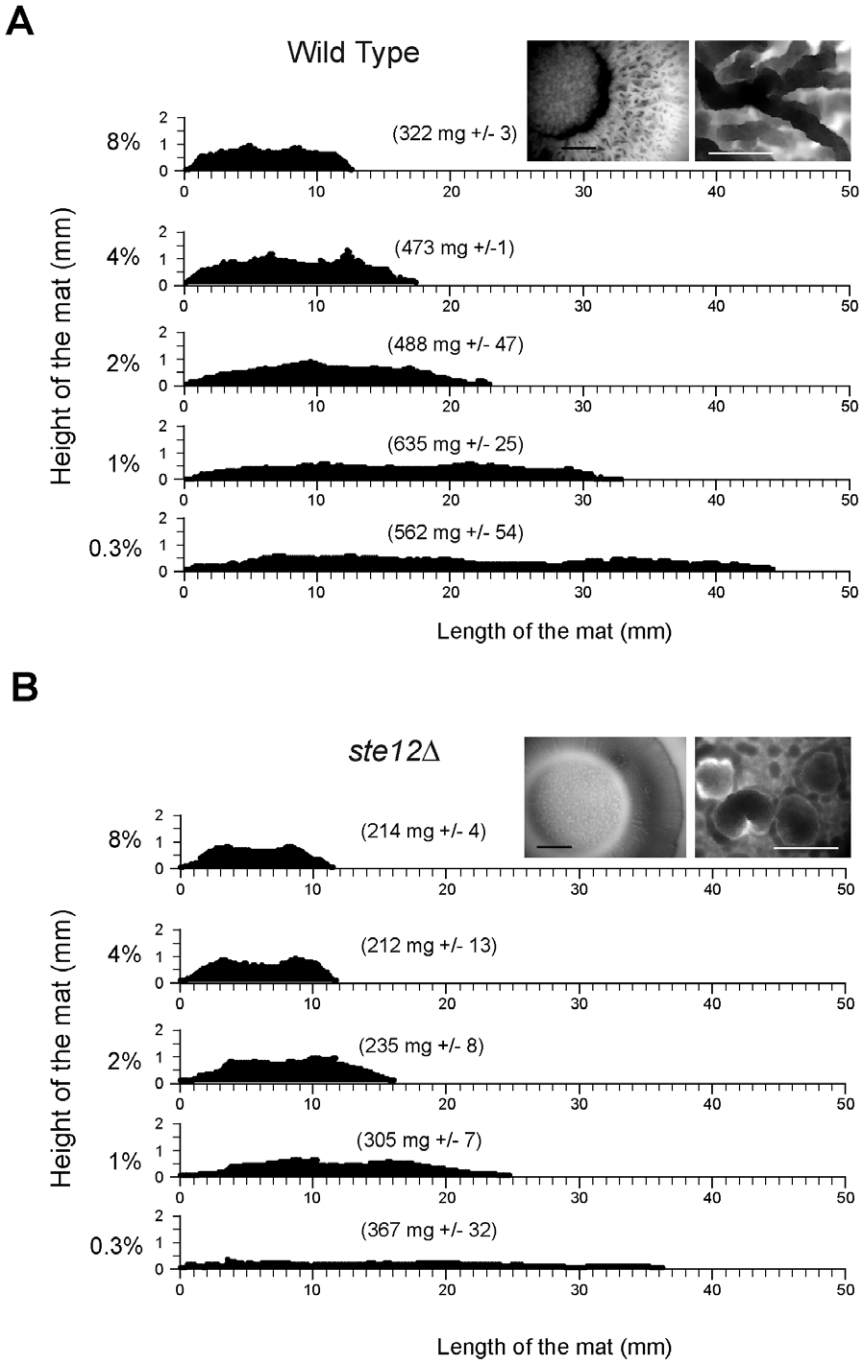
The role of the MAPK pathway in regulating mat architecture when exposed to surfaces of different rigidities. **A**) Contour maps in the Z-axis of wild type (PC538) mats incubated in media of different agar concentrations for 14d. Insets show mat morphology (left, photograph, bar, 1 cm; right, photomicrograph, bar, 200 microns) in 4% agar. The numbers in parentheses represent the average mat dry weight from two experiments with standard deviation shown. Scale bars for the X and Y-axes are in mm. **B**) Mats formed by a *ste12*Δ mutant (PC539) on different agar concentrations. Analysis is as described for panel A.

**Figure 5:**
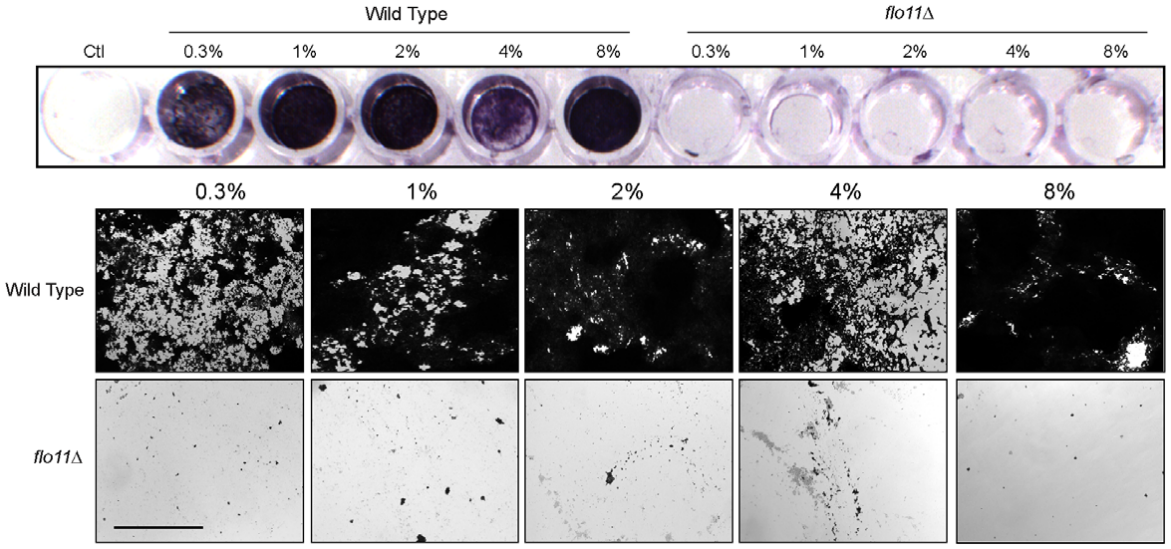
Yeast mats grown on a variety of surface rigidities exhibit Flo11-dependent adherence. Mats of wild type (PC 538) and *flo11*Δ (PC 1029) mutant were grown for 11 days on YEPD medium containing % agar concentrations as indicated. Adherence of cells in different mats was estimated by adherence to plastic using crystal violet dye. The control well (Ctl) contained water only. Below, photomicrographs of the wells are shown. Top panels, wild type; bottom panels, *flo11*Δ. Agar concentrations are as indicated. Bar = 10 µm.

In line with a previous report [53], we found that mats grew upward in the plane of the Z-axis in response to increasing surface rigidity (Fig. 4A). Mats grown on high agar concentrations were taller, more compact, and somewhat less massive (Fig. 4A). An implication of these results is that mats - which have previously been studied on low-agar medium (0.3% agar) - also form on media of different agar concentrations. Cells derived from low- and high-agar mats showed Flo11-dependent adherence to plastic (Fig. 5), which confirmed that mats formed on high-agar media (1%, 2%, 4%, and 8% agar) are representative of those characterized on low-agar media (0.3%). Mats grown on high agar medium were architecturally complex (Fig. 4A). Upward growth in the plane of the Z-axis was partially dependent on the MAPK pathway (Fig. 4B, *ste12*Δ) and Flo11 (Fig. S1B). Some upward growth occurred in a MAPK-independent manner, which indicates that other regulatory pathways might also contribute to the response. Some of the increase in vertical growth might also be a consequence of physical restraints as posed by dryness of the substratum.

**Figure 6:**
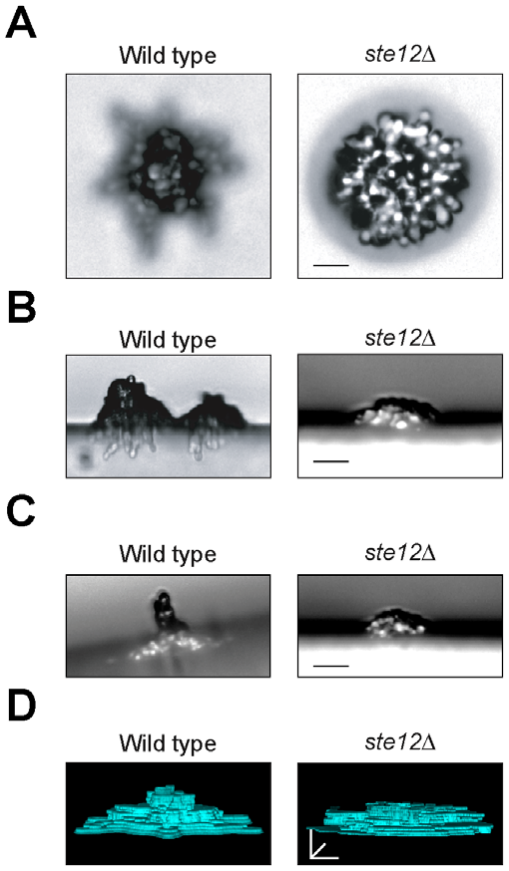
Analysis of filamentation in mats grown on high-agar surfaces. Wild type (PC538) and *ste12*Δ mutant (PC539) were compared by microscopy at 10× on 4% agar medium. **A**) Top view of typical microcolonies grown on synthetic medium lacking glucose with 4% agar, Bar, 20 microns. **B**) Cross-section view of typical microcolonies as those described in panel 6A. Images were obtained by cutting the agar medium and laying agar slabs at a 90° angle. Bar, 20 microns. **C**) Plates tilted at a 20° angle. Shown are examples of arial pseudohyphae produced by wild type microcolonies (PC538). At right, the *ste12*Δ (PC539) mutant fails to form extensive arial pseudohyphae. The image shown is a compiled Z-stack rendering of a typical microcolony. Bar = 25 microns. D) 3D rendering of the microcolonies shown in panel A. Movies of the rendered images are found in the supplemental materials (Supplemental Movies S1, S2, S3, and S4).

The filamentous growth pathway might regulate upward growth by the formation of pseudohyphae and/or by regulating *FLO11* expression. To assess the contribution of pseudohyphae in upward growth, microcolonies were examined on low nutrient (-Glu), high agar (4%) medium. In this setting, microcolonies grew upward in a conical tube of cells atop a pseudohyphal base (Fig. 6A–C wild type and Supplemental Movie S3). Cells in contact with the agar surface were filamentous (Fig. 6B, >95% of all cells), whereas cells that comprised the cone were a mixture of yeast- and filamentous-form cells (Fig. 6B and 6C, ∼50% each cell type). Therefore, pseudohyphal cells constitute a specialized component of upwardly growing mats that functionally resemble an adhesive, invasive and expanding “foot”. The filamentous growth pathway was required for pseudohyphal formation on low nutrient, high agar medium (Fig. 6A–C *ste12*Δ, Supplemental Movie S4), to produce the aerial pseudohyphae (Fig. 6B and C), and for microcolony architecture (Fig. 6D). Therefore, the MAPK pathway controls mat height and architecture across a range of agar concentrations in part by regulating the formation of pseudohyphae.

**Figure 7:**
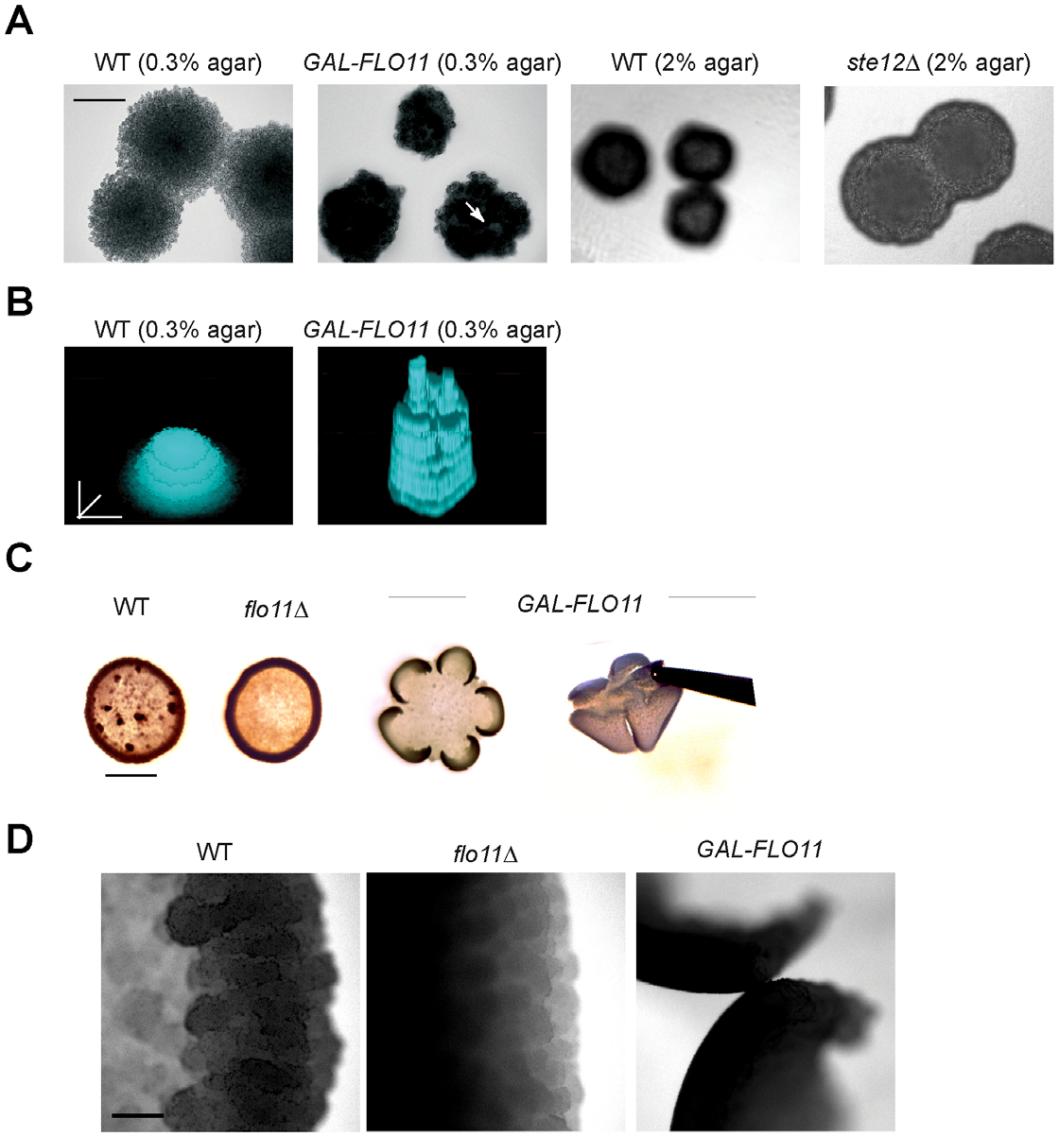
The role of Flo11 overexpression on upward growth in the plane of the Z-axis. **A**) Microcolonies of wild-type cells (PC538) and cells overexpressing *FLO11* (PC2712) were examined by microscopy at 10× after 24 h incubation at 30°C. Wild type and *ste12* mats on high agar concentrations is also shown Bar = 100 microns. **B**) Contour mapping of z-stack rendering of the indicated microcolonies in panel 7A are shown. Bar = 30 microns. See Supplemental Movies S5 and S6. **C**) Wild-type (PC538), *flo11*Δ (PC1029), and *GAL-FLO11* (PC2712) cells were spotted onto YEP-GAL medium (8%) agar atop nitrocellulose filters for 24 h at 30°C. Colonies were photographed in transmitted light. Bar = 1 cm. At right, separation of the *GAL-FLO11* mat from the surface using forceps. **D**) Microscopic examination of the mats in panel C. Bar = 200 microns.

Since much of the contribution to mat structure from MAPK pathway requires Flo11, the effect of overexpression of *FLO11* on mat architecture was examined. Overexpression of *FLO11* induced highly structured microcolonies with a ruffled appearance and non-uniform perimeters (*GAL-FLO11*, Fig. 7A, arrow; Supplemental Movies S1 and S2). The overexpression of *FLO11* on low-agar medium resembled the growth of wild-type mats grown on high-agar medium [Fig. 7A, WT (high agar); Supplemental Movie S5]. Cells lacking an intact MAPK pathway, which fail to express *FLO11*, failed to show this pattern [Fig. 7A, *ste12*Δ (high agar); Supplemental Movie S6].

Under conditions favorable for upward growth (-Glu, 4% agar), *FLO11* overexpression (*GAL-FLO11*) induced the formation of brittle mats that peeled away from the surface (Fig. 7C) and inhibited mat expansion. These mats were separable from the surface by forceps (Fig. 7C far right panel), which is indicative of an unprecedented degree of Flo11-mediated cell-cell adhesion. Microscopic examination showed that *GAL-FLO11* mats were highly compact compared to wild-type mats grown under the same condition (Fig. 7D). Therefore, the cell-cell adherence properties of Flo11 can remodel the behavior of a mat.

**Figure 8:**
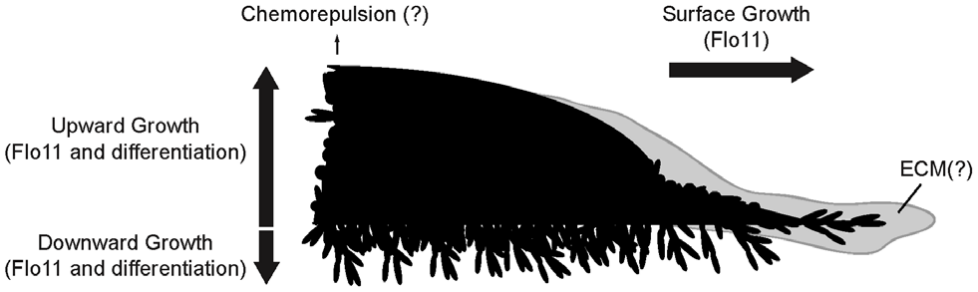
Model for the different mat responses controlled by the MAPK pathway. Schematic of a mat expanding under nutrient-limiting conditions is shown. Different responses regulated by the MAPK pathway may include: 1) mat expansion in the plane of the XY-axis (surface growth, through Flo11 [23]), 2) cell differentiation that causes invasive growth in the Z-axis (downward growth, Flo11 and differentiation), and 3) upward growth in the plane of the Z-axis in response to surface rigidity and nutrient-limiting conditions (Flo11 and differentiation). This upward growth may represent a type of chemorepulsion. An extracellular matrix (ECM), which may contain shed Flo11 [48] as well as other proteins is depicted.

In conclusion, we show that two nutritional scavenging responses in yeast, filamentous growth and mat formation, which have been typically studied under separate contexts, are part of an integrated behavior. We also show that mats can engage in an upward growth pattern in response to nutrient limitation and surface rigidity. The MAPK pathway regulates these interrelated responses through the formation of pseudohyphae and by regulating the expression of the *FLO11* gene. In this way, the filamentous growth pathway may function to regulate mat behaviors in response to a variety of extracellular stresses (Fig. 8). One consequence of colonial remodeling is that mats may regulate their expansion and undergo chemorepulsive behaviors (Fig. 8). In pathogens, MAPK pathways may have a broader role in regulating fungal social behaviors than is currently appreciated.

## Materials and Methods

### Strains, Plasmids, and Microbiological Techniques

Yeast and bacterial strains were manipulated by standard methods [54], [55]. Yeast parental strains in the ∑1278b background, PC313 (*MATa ura3-52*) and PC 538 (*MAT* **a** *ste4 FUS1-lacZ FUS1-HIS3 ura3-52*) were described previously [35]. PC549, PC948, PC1083 and PC2382 were made as described in [35]. PC1029, PC2712 and PC2716 were generated as explained in [48]. PC3039 was made as listed in [46]. PC2670 (PC948 *flo11:URA3*) and PC2717 (PC2712 *msb2:URA3*) were generated using standard protocols. Gene disruptions and *GAL1* promoter fusions were made by PCR-based methods [56], [57], including the use of antibiotic resistant markers [58]. Integrations were confirmed by PCR analysis and phenotype. Budding pattern was based on established methodology [59], and by visual inspection as described [47]. The single-cell invasive growth assay [30] and plate-washing assay [32] were performed as published. Differences between Sigma1278b strains may also account for reported differences in mat responses. Pseudohyphae were also observed in mat interiors albeit at a lower level than perimeters. Mats were weighed by scraping cells off of the surface of plates into water followed by centrifugation. Experiments were performed in duplicate and standard deviation between trials is shown. Residual carbon sources allowed for growth in synthetic and rich media not supplemented with glucose [30]. Slightly different conditions were used for nutrient limiting conditions (-GLU), depending on the levels of residual carbon sources in the media. This allowed for evaluation of the tested phenotypes. The terms filament and pseudohyphae are used interchangeably.

### Microbial Mat Assays

Mats were grown as previously described [23] on media containing 0.3% agar unless indicated otherwise. Variegation in *FLO11* expression [60] cause slight changes in mat expansion properties resulting in outgrowths from a regularly expanding mat.

### Plastic adherence assay

This assay was adapted from Reynolds and Fink 2001. Mats were grown on YEPD media containing 0.3, 1, 2, 4, or 8% agar for 11 days to determine mat properties on different agar concentrations. Cells were removed from mats using a toothpick, resuspended in water, and adjusted to an optical density of A_600_ = 2.0. One hundred microliter aliquots of cell suspensions were added to polystyrene wells (Falcon Microtest Tissue culture plate, 96 Well) and incubated for 4 h. An equal volume of 1% crystal violet dye (DIFCO) was added to each well for 20 min. Wells were washed 5 times, photographed and adherent cells were visualized by microscopy at 10×.

### Microscopy

Differential-interference-contrast (DIC) was performed using an Axioplan 2 fluorescent microscope (Zeiss) with a PLAN-APOCHROMAT 100X/1.4 (oil) objective (N.A. 0.17). Digital images were obtained with the Axiocam MRm camera (Zeiss). Axiovision 4.4 software (Zeiss) was used for image acquisition and analysis and for rendering 3D Z-stack images. Contour mapping of mat surfaces was performed by measuring the height in microns of mats, recorded as Z-stack images by DIC microscopy. Images were further analyzed in Adobe Photoshop, where adjustments of brightness and contrast were made.

### Computational Analysis

To generate 3D colony and microcolony models, cells in the focal plane for each Z-stack image were selected and converted into a stack in ImageJ. Image properties were adjusted corresponding to the height, width, and depth (voxel depth) for each colony. Models were generated using the 3D Viewer plugin with a volume rendering and a resampling factor of 1. To manually generate 3D movies of colonies, images were generated of the model at Y-rotational angles using the Apply Transform function in the ImageJ 3D Viewer. Transformation matrices were applied to rotate and scale the model. Images generated through this process were used to generate an animated GIF file. Other movies were generated using the animation and record functions of the ImageJ 3D viewer.

## Supporting Information

Figure S1 **Contribution of MAPK pathway to mat expansion and/or invasion on different agar concentrations.** **A**) Wild type (PC538), *flo11*Δ (PC1029), and *ste12*Δ (PC2382). Mats were grown for 4d at 30°C on YEPD+1.0% agar medium and washed in a stream of water to reveal invaded cells. Bar = 1 cm. **B**) Wild type (PC538), *ste20*Δ (PC540), and *flo11*Δ (PC1029) on YEPD for 20d at 30°C. Bar = 1 cm.
(TIF)

Supplemental Movie S1 **The effect of overexpression of** ***FLO11*** **on microcolony morphology: wild-type control.** Example of a wild type microcolony (PC538) is shown. The animation was created from a 3D rendering using ImageJ 3D Viewer. Z-stack images were taken at 7 µm intervals at 5× magnification of a typical colony grown for 48 h at 30° on YEP-GAL medium (0.3% agar). The microcolony has an approximate height of 28 µm.
(AVI)

Supplemental Movie S2 **The effect of overexpression of** ***FLO11*** **on microcolony morphology:** ***GAL-FLO11*** **.** Example of a colony overexpressing *FLO11* (*GAL-FLO11* [PC2712]) is shown. The animation was created from a 3D rendering using ImageJ 3D Viewer. Z-stack images were taken at 7 µm intervals at 5× magnification of colonies that were grown for 48 h at 30° on YEP-GAL medium (0.3% agar). The microcolony has an approximate height of 70 µm.
(AVI)

Supplemental Movie S3 **Architecture of a wild-type microcolony grown on nutrient-limiting medium.** Animation was created from 3D renderings using ImageJ 3D Viewer. The original images were taken from cells incubated for 24 h on SC medium agar. Z-stacks were taken in 1.73 µM increments. The colony is approximately 26 µm tall.
(AVI)

Supplemental Movie S4 **Architecture of a** ***ste12*** **Δ mutant microcolony grown on nutrient-limiting medium.** Animation was created from 3D renderings using ImageJ 3D Viewer. The original images were taken from cells incubated for 24 h on SC medium agar. Z-stacks were taken in 1.73 µM increments. The *ste12*Δ colony 17 µm tall.
(AVI)

Supplemental Movie S5 **Z-stack image of wild-type microcolonies grown on 4% agar medium containing glucose as a carbon source (SD).**
(MOV)

Supplemental Movie S6 **Z-stack image of** ***ste12*** **Δ microcolonies grown on 4% agar medium containing glucose as a carbon source (SD).**
(MOV)
