# Supplementary figures and images for: Regulation of Mat Responses by a Differentiation MAPK Pathway in Saccharomyces cerevisiae
Source: PLoS One. 2012 Apr 4;7(4):e32294. doi: 10.1371/journal.pone.0032294 (PMC3319557; doi:10.1371/journal.pone.0032294)

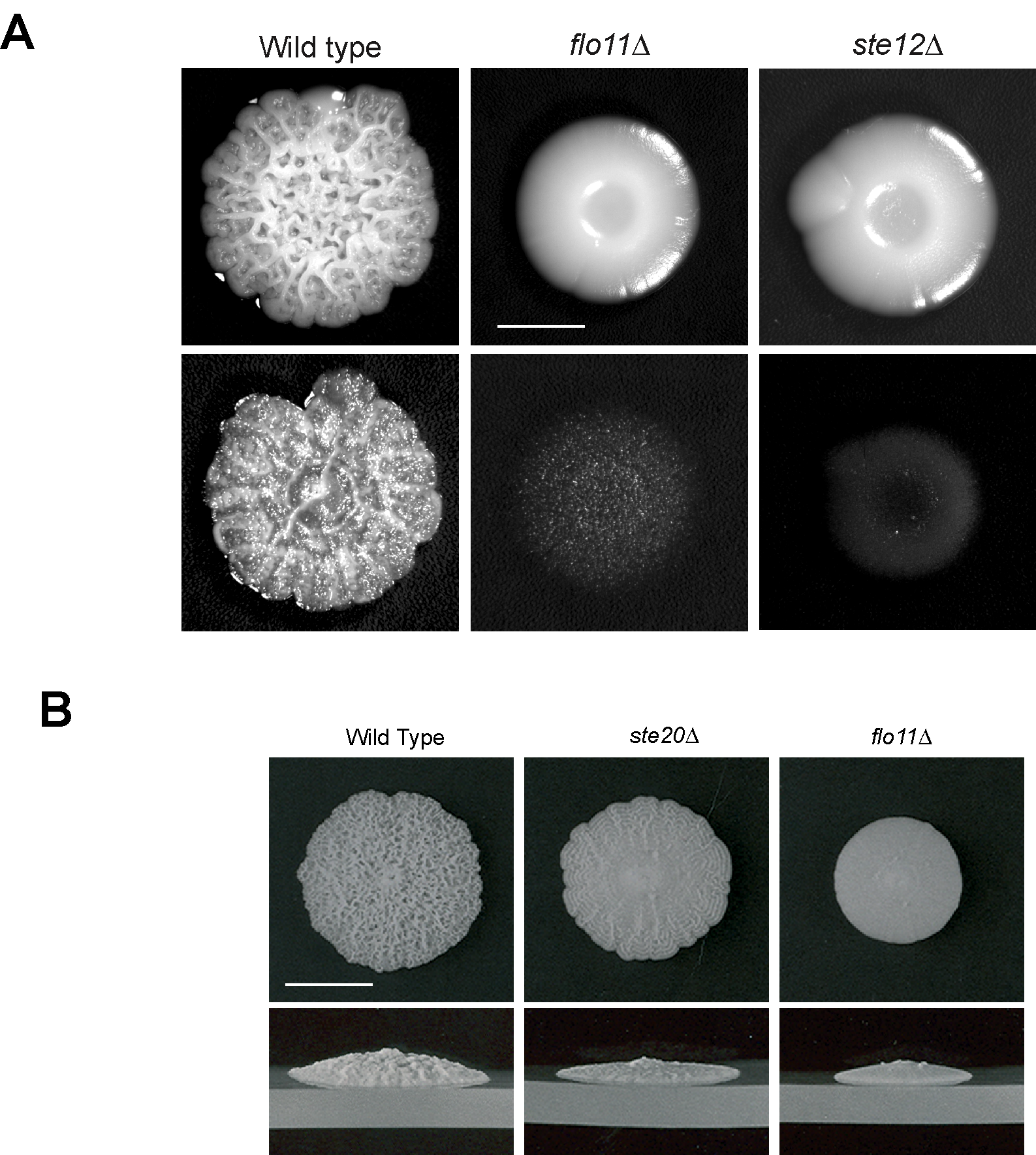

Supplement: Figure S1 — Contribution of MAPK pathway to mat expansion and/or invasion on different agar concentrations. A) Wild type (PC538), flo11Δ (PC1029), and ste12Δ (PC2382). Mats were grown for 4d at 30°C on YEPD+1.0% agar medium and washed in a stream of water to reveal invaded cells. Bar = 1 cm. B) Wild type (PC538), ste20Δ (PC540), and flo11Δ (PC1029) on YEPD for 20d at 30°C. Bar = 1 cm. (TIF) [file pone.0032294.s001.tif]
